# Cooperation of Sox4 with β-catenin/p300 complex in transcriptional regulation of the *Slug* gene during divergent sarcomatous differentiation in uterine carcinosarcoma

**DOI:** 10.1186/s12885-016-2090-y

**Published:** 2016-02-03

**Authors:** Hisako Inoue, Hiroyuki Takahashi, Miki Hashimura, Koji Eshima, Masashi Akiya, Toshihide Matsumoto, Makoto Saegusa

**Affiliations:** Department of Pathology, 1-15-1 Kitasato, Minami-ku, Sagamihara, Kanagawa 252-0374 Japan; Department of Immunology, Kitasato University School of Medicine, 1-15-1 Kitasato, Minami-ku, Sagamihara, Kanagawa 252-0374 Japan

**Keywords:** Sox, β-catenin, Slug, p300, Uterine carcinosarcoma

## Abstract

**Background:**

Uterine carcinosarcoma (UCS) represents a true example of cancer associated with epithelial-mesenchymal transition (EMT), which exhibits cancer stem cell (CSC)-like traits. Both Sox and β-catenin signal transductions play key roles in the regulation of EMT/CSC properties, but little is known about their involvement in UCS tumorigenesis. Herein, we focused on the functional roles of the Sox/β-catenin pathway in UCSs.

**Methods:**

EMT/CSC tests and transfection experiments were carried out using three endometrial carcinoma (Em Ca) cell lines. Immunohistochemical investigation was also applied for a total of 32 UCSs.

**Results:**

Em Ca cells cultured in STK2, a serum-free medium for mesenchymal stem cells, underwent changes in morphology toward an EMT appearance through downregulation of E-cadherin, along with upregulation of *Slug*, known as a target gene of β-catenin. The cells also showed CSC properties with an increase in the aldehyde dehydrogenase (ALDH) 1^high^ activity population and spheroid formation, as well as upregulation of Sox4, Sox7, and Sox9. Of these Sox factors, overexpression of Sox4 dramatically led to transactivation of the *Slug* promoter, and the effects were further enhanced by cotransfection of Sox7 or Sox9. Sox4 was also able to promote β-catenin-mediated transcription of the *Slug* gene through formation of transcriptional complexes with β-catenin and p300, independent of TCF4 status. In clinical samples, both nuclear β-catenin and Slug scores were significantly higher in the sarcomatous elements as compared to carcinomatous components in UCSs, and were positively correlated with Sox4, Sox7, and Sox9 scores.

**Conclusions:**

These findings suggested that Sox4, as well as Sox7 and Sox9, may contribute to regulation of EMT/CSC properties to promote development of sarcomatous components in UCSs through transcriptional regulation of the *Slug* gene by cooperating with the β-catenin/p300 signal pathway.

**Electronic supplementary material:**

The online version of this article (doi:10.1186/s12885-016-2090-y) contains supplementary material, which is available to authorized users.

## Background

Uterine carcinosarcomas (UCSs), previously referred to as malignant mixed mullerian tumors, are aggressive neoplasms that contain both carcinomatous and sarcomatous elements, and the incidence is only 2–5 % of all uterine carcinomas [1, 2]. Clinically, more than 40 % of patients with UCSs are categorized as advanced stage at diagnosis, and over 50 % of cases show recurrence of the disease [3]. Histopathologically, the most common epithelial components are serous, followed by endometrioid type, while the sarcomatous components are composed of homologous (composed of tissues normally found in the uterus) or heterologous tissues (containing tissues not normally found in the uterus, most commonly malignant cartilage and skeletal muscle) [4, 5].

Epithelial-mesenchymal transition (EMT) plays a central role in converting both normal and neoplastic epithelial cells into derivatives with a more mesenchymal phenotype [6, 7]. A hallmark of EMT is loss of cell-cell adhesion molecules, down-regulation of epithelial differentiation markers, and transcriptional induction of mesenchymal markers, along with nuclear localization of β-catenin [8]. Snail, Slug, and Twist, all repressors of the *E-cadherin* gene, are also involved in the process [9–12]. Given that UCSs are regarded as metaplastic carcinomas when the sarcomatous component is derived from the carcinoma, it is suggested that EMT may play an important role in tumorigenesis of UCSs.

A growing body of evidence shows that tumors contain a very small subpopulation of cancer stem cells (CSCs) or tumor-initiating cells [13]. CSCs, similar to somatic stem cells, are defined as cells within a tumor that possess the capacity to self-renew and to differentiate into the heterogeneous lineages of cancer cells that comprise the tumors [14]. Interestingly, a relationship between EMT and CSCs has been proposed with evidence demonstrating that EMT cells exhibit stem cell-like traits and CSCs acquire mesenchymal-like characteristics, [14] pointing to the possibility that sarcomatous stem-like cells derived from carcinoma cells may also be present and act as progenitors for divergent sarcomatous differentiation.

Both Sox and β-catenin signal transductions display a broad spectrum of biological function in the regulation of EMT/CSC properties in a wide variety of cells [15–17]. We therefore hypothesize that this signal pathway may contribute to the determination of phenotypic characteristics through modulation of EMT/CSC properties in UCSs. To test this, we hereby investigated the expression of several Sox factors, β-catenin, and Slug, with reference to EMT/CSC properties, using endometrial carcinoma (EmCa) cell lines and clinical UCS samples.

## Methods

### Plasmids and cell lines

The pGL3B-Slug luc constructs, including −2125/−235 bp, −1859/−235 bp, −1587/−235 bp, and −813/−235 bp fragments, pcDNA3.1-HA-β-cateninΔS45, pcDNA3.1-Sox4, pcDNA3.1-Sox7, pcDNA3.1-Sox9, pcDNA3.1-HA-Slug, PCI-Flag-p300, pcDNA3.1-TCF4ΔN30 (dominant-negative form of TCF4), pG5 luc, and pM-β-cateninΔS45 were used as described previously [18–21]. pM-Sox4 was constructed by inserting the Sox4 cDNA fragment into the pM DNA-BD vector (BD Biosciences Clontech, Worcester, MA, USA). Site-directed mutagenesis of putative Sox4 binding sites in the *Slug* promoter was performed using the PrimeSTAR Mutagenesis Basal kit (Takara Bio, Shiga, Japan).

The Em Ca cell lines, Ishikawa, Hec251, and Hec6 cells, were maintained in Eagle’s MEM with 10 % bovine calf serum. To establish cells stably overexpressing HA-Slug, the expression plasmids or empty vectors were transfected into Hec6 cells, and stable clones were established as described previously [20].

### Antibodies and reagents

Anti-β-catenin and anti-p27^kip1^ antibodies were purchased from BD Biosciences (San Jose, CA, USA). Anti-Sox4, anti-Sox6, anti-Sox7, anti-Sox9, anti-Sox11, and β-actin antibodies were obtained from Sigma-Aldrich Chemicals (St. Louis, MO, USA). Anti-Snail and anti-Slug antibodies were from Cell Signaling (Danvers, MA, USA). Anti-p21^waf1^, anti-cyclin D1, and anti-CD44s antibodies were purchased from Dako (Copenhagen, Denmark). Anti-Sox2 and anti-cyclin A antibodies were from Abcam (Cambridge, MA, USA) and Novocastra (Newcastle, UK), respectively. Anti-HA and anti-E-cadherin antibodies were obtained from Santa Cruz Biotechnology (Santa Cruz, CA, USA) and Takara (Shiga, Japan) respectively. Anti-CD133 antibody was from Miltenyi Biotechnology (Bergisch Gladbach, Germany).

STK2, which is a serum-free culture medium for mesenchymal stem cells, [22] was obtained from DS Pharma Biomedical (Osaka, Japan).

### Transfection

Transfection was carried out using LipofectAMINE PLUS (Invitrogen, Carlsbad, CA, USA) in duplicate or triplicate as described previously [18–21]. Luciferase activity was assayed as described previously [18–21].

### Real-time reverse-transcription polymerase chain reaction (RT-PCR)

cDNA was synthesized from 2 μg of total RNA. For quantitative analysis, real-time RT-PCR was carried out using a Power SYBR Green PCR Master Mix (Applied Biosystems, Foster City, CA, USA) with specific primers (Table 1). Fluorescent signals were detected using the ABI 7500 real-time PCR System, and data were analyzed using the associated ABI 7500 System SDS Software (Applied Biosystems). Primers for the *GAPDH* gene were also applied, as described previously [18–21].

**Table 1:** Primer sequences used in the study

Assay	Gene/region		Sequence	Size
Mutagenesis	Slug/Sox4-M1	Forward	5′-ACTTTTAGGGGTTGTGGATAGACTGTGT-3′	
		Reverse	5′-ACAACCCCTAAAAGTGTTAGACAATGT-3′	
	Slug/Sox4-M2	Forward	5′-AGGATTAGGGTGAATTATTTTCTCTGTT-3′	
		Reverse	5′-AATTCACCCTAATCCTTATGCTAATGGA-3′	
	Slug/Sox4-M3	Forward	5′-AATAATAGGGGAAATTAGCTTAGGAAAT-3′	
		Reverse	5′-ATTTCCCCTATTATTCTTATTTCTTCC-3′	
	Slug/Sox4-M4	Forward	5′-GAGGGCAGGGAAGCATTTCTTTCAAGCC-3′	
		Reverse	5′-TGCTTCCCTGCCCTCTAAAGGCAGGCT-3′	
ChIP	Slug/Sox4-1	Forward	5′-GTGTTATAACTACCAGCAAA-3′	132 bp
		Reverse	5′-ACAAATATAGCACAGTTGAG-3′	
	Slug/Sox4-2	Forward	5′-TCTCCTGCAAGTACAGTTCC-3′	149 bp
		Reverse	5′-TGTTTGGAGGGTGAGGTGG-3′	
	Slug/Sox4-3	Forward	5′-AGTGACTGTTGGAAGAAATA-3′	141 bp
		Reverse	5′-AAAGTGCATTGTCAGGTTG-3′	
	Slug/Sox4-4	Forward	5′-TCAGCCTGCCTTTAGAGGGC-3′	121 bp
		Reverse	5′-GCTACTCAGGGCTTCCGCG-3′	
mRNA	Slug	Forward	5′-ACGCAATCAATGTTTACTCG-3′	277 bp
		Reverse	5′-TGAAGAGAAAGGTTACTGTC-3′	
	E-Cadherin	Forward	5′-CAACATGGGAGGTGAGAGTTT-3′	319 bp
		Reverse	5′-CGAAGAAACAGCAAGAGCAGCAGAATCAGA-3′	
	Sox4	Forward	5′-GTTCGGCGTGTGCTTGGC-3′	261 bp
		Reverse	5′-GTCTTGCACCAGCTCGGG-3′	
	Sox7	Forward	5′-AAGCCCTCTCCACTGTAGCC-3′	245 bp
		Reverse	5′-TTGCGATCCATGTCCCCCAG-3′	
	Sox9	Forward	5′-CAGCAAGAACAAGCCGCACG-3′	222 bp
		Reverse	5′-GTAATCCGGGTGGTCCTTCTT-3′	

### Western blot assay and immunoprecipitation

Total cellular proteins were isolated using RIPA buffer [20 mM Tris–HCl (pH7.2), 1 % Nonidet P-40, 0.5 % sodium deoxycholate, 0.1 % sodium dodecyl sulfate]. Aliquots of the proteins were resolved by SDS-PAGE, transferred to membranes, and probed with primary antibodies, coupled with the ECL detection system (Amersham Pharmacia Biotechnology, Tokyo, Japan).

For immunoprecipitation, cells cultured in STK2 were lysed with TNE buffer [10 mM Tris–HCl (pH7.6), 150 mM NaCl, 1 % NP-40, 1 mM EDTA]. Cell lysates were cleared and incubated with anti-Sox4 antibody, followed by incubation with Protein G-Sepharose (Amersham Pharmacia Biotechnology). Western blot assay was subsequently performed with anti-β-catenin and anti-Sox4 antibodies.

### Flow cytometry and Aldefluor assay

Cells were fixed using 70 % alcohol and stained with propidium iodide (Sigma) for cell cycle analysis. ALDH 1 enzyme activity in viable cells was determined using a fluorogenic dye based Aldefluor assay (Stem Cell Technologies, Grenoble, France) according to the manufacturer’s instructions. The prepared cells were analyzed by flow cytometry using BD FACS Calibur (BD Biosciences) and CellQuest Pro software version 3.3 (BD Biosciences).

### Spheroid assay

Cells (x10^3^) were plated in low cell binding plates (Thermo Fisher Scientific, Yokohama, Japan) in STK2 or Eagle’s MEM with 10 % bovine calf serum. Uniform spheroids of at least 50 μm in size were counted approximately 2 weeks after plating.

### Chromatin immunoprecipitation (ChlP) assay

ChIP analysis was performed using an EpiXplore ChIP assay kit (Clontech Laboratory, Mountain View, CA, USA). Briefly, after culture in STK2 for 1 week, cells were cross-linked with formaldehyde. Cell lysates were sonicated to shear DNA to lengths between 200 and 1000 bp, and then precipitated overnight using anti-Sox4 antibody or rabbit IgG as negative control, along with magnetic beads. After proteinase K digestion, DNA was extracted and analyzed by PCR. ChIP analysis was conducted with a reduction in the number of cycles from 30 to 25, using four specific primer sets.

### Clinical cases

We reviewed cases of comprehensively staged high-grade endometrial adenocarcinomas from the patient records of Kitasato University Hospital in the period from 1997 to 2012. According to the criteria of the 2014 World Health Organization classification, [23] tumors were designated as UCS if they had evidence of both malignant epithelial (endometrioid, serous, or clear cell) components and mesenchymal (homologous or heterologous) elements. Endometrioid adenocarcinomas with spindle elements and hyalinized stroma were specifically excluded. Finally, a total of 32 UCSs were investigated. Of these, 9 cases had endometrioid and 23 cases had non-endometrioid epithelial components, while 25 and 8 cases showed homologous and heterologous mesenchymal elements, respectively. All tissues were routinely fixed in 10 % formalin and processed for embedding in paraffin wax. Approval for this study was given by the Ethics Committee of the Kitasato University School of Medicine (B14-35). Signed informed consent forms were not required from the participants due to the retrospective approach of the study, which did not impact on their treatment.

### Immunohistochemistry (IHC)

IHC was performed using a combination of the microwave-oven heating and polymer immunocomplex (Envision, Dako) methods, as described previously [18–21]. The immunoreactions were visualized with DAB (3,3′ diaminobenzidine), and the nuclei were counterstained with methyl green.

For evaluation of IHC findings, scoring of nuclear immunoreactivity was performed, on the basis of the percentage of immunopositive cells and the immunointensity, with multiplication of values of the two parameters, as described previously [18–21].

### Statistics

Comparative data were analyzed using the Mann–Whitney *U*-test, and the Spearman’s correlation coefficient. The cutoff for statistical significance was set as *p* < 0.05.

## Results

### Changes in expression of Sox factors during the EMT process in Em Ca cells

**Fig. 1:**
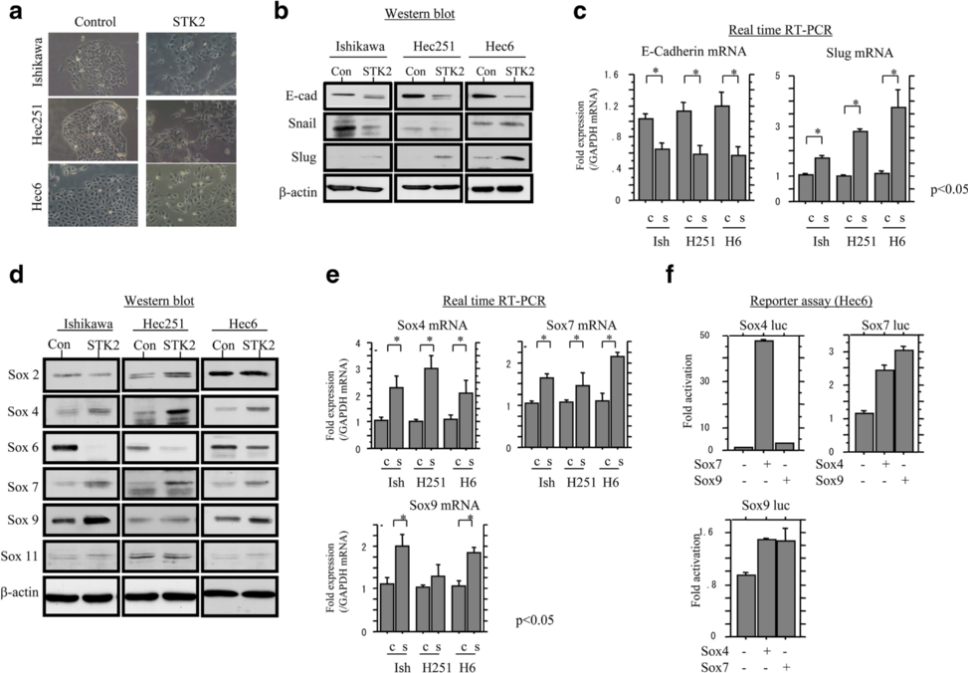
Changes in expression of Sox factors during the EMT process in Em Ca cells. **a** Phase-contrast images of Ishikawa, Hec251, and Hec6 cells cultured in STK2 for 5 days. Western blot analysis (**b**) and real time RT-PCR (**c**) for the indicated molecules from Ishikawa, Hec251, and Hec6 cells cultured in STK2. Con and c, control; s, STK2; Ish, Ishikawa cells. **d** Western blot analysis for the indicated proteins from Ishikawa, Hec251, and Hec6 cells cultured in STK2. Con, control. **e** Real time RT-PCR for the indicated mRNA transcripts from Ishikawa, Hec251, and Hec6 cells cultured in STK2. c, control; s, STK2. **f** Hec6 cells were transfected with Sox4, Sox7, and Sox9 reporter constructs, together with either Sox4, Sox7, or Sox9. Relative activity was determined based on arbitrary light units of luciferase activity normalized to pRL-TK activity. The activities of the reporter plus the effector relative to that of the reporter plus empty vector are shown as means ± SDs. The experiment was performed in duplicate

To induce EMT in Em Ca cells, the three cell lines, including Ishikawa, Hec251, and Hec6 cells, were cultured in STK2, a serum-free medium for mesenchymal stem cells [22]. As shown in Fig. 1a, cells cultured in STK2 demonstrated a dramatically altered morphology toward a fibroblast-like appearance after 73 h, along with decreased E-cadherin and increased Slug expression at both mRNA and protein levels (Fig. 1b and c). In contrast, changes in Snail expression at the protein level (Fig. 1b), as well as the mRNA level, were relatively weak (data not shown).

Next, we examined whether Sox factors are directly involved in regulation of the EMT process observed in cells cultured in STK2, since some molecules are involved in the promotion of EMT [15]. The Ishikawa, Hec251, and Hec6 cells cultured in STK2 showed increased expression of Sox4, Sox7, and Sox9, but not Sox6 and Sox11, at both protein and mRNA levels (Fig. 1d and e). In addition, the *Sox4* promoter activity was increased by 20–50 folds following transfection of Sox7, while changes in the promoter activity of both *Sox7* and *Sox9* in response to other Sox factors were relatively minor (Fig. 1f and Additional file 1: Figure S1A). These findings suggested that culturing Em Ca cells in STK2 was sufficient to induce EMT, along with downregulation of E-cadherin and upregulation of Slug. In addition, upregulation of some Sox factors through formation of complex transcriptional regulatory loops occurs during the EMT process in Em Ca cells.

### Relationship between EMT and CSC properties in Em Ca cells

**Fig. 2:**
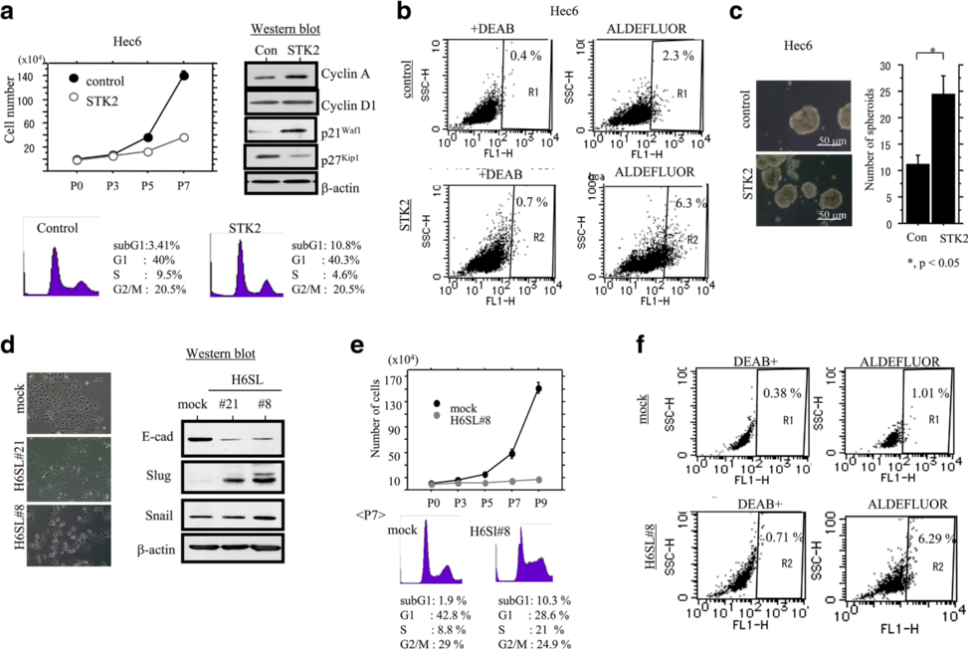
Association between Slug expression and EMT/CSC properties in Em Ca cells. **a** Upper left: Hec6 cells were seeded at low density with or without STK2. The cell numbers are presented as means ± SDs. P0, P3, P5, and P7 indicate 0, 3, 5, and 7 days after cell passage, respectively. Upper right: western blot analysis for the indicated proteins from Hec6 cells cultured in STK2. Lower: cell cycle analysis of Hec6 cells cultured in STK2 for 6 days by flow cytometry. Con, control. **b** Aldefluor analysis of Hec6 cells cultured in STK2 (lower) and its control (upper) for 5 days. Cells negative for ALDH activity (treated with ALDH inhibitor DEAB) are located in the area to the far left of each plot, and the positive cells are in black gates (R1 and R2). The percentage of live single cell population contained in each gate is shown. DEAB, diethylaminobenzaldehyde. **c** *Left*: phase-contrast images of spheroids of Hec6 cells cultured in STK2 for 2 weeks. *Right*: the number of spheroids is presented as means ± SDs. Con, control. **d** *Left*: phase-contrast images of two independent Hec6 cell lines stably overexpressing Slug (H6SL#8 and #21) and mock-transfected cells. *Right*: western blot analysis for the indicated proteins from H6SL#8 and #21 cells and the mock. **e** *Upper*: H6SL#8 cells were seeded at low density. The cell numbers are presented as means ± SDs. P0, P3, P5, P7, and P9 indicate 0, 3, 5, 7, and 9 days after cell passage, respectively. *Lower*: cell cycle analysis of H6SL#8 and mock cells at 7 days by flow cytometry. **f** Aldefluor analysis of H6SL#8 (lower) and mock (upper) cells. Cells negative for ALDH activity (treated with ALDH inhibitor DEAB) are located in the area to the far left of each plot, and the positive cells are in black gates (R1 and R2). The percentage of live single cell population contained in each gate is shown. DEAB, diethylaminobenzaldehyde

To examine whether EMT is linked to CSC properties, Hec6 cells were selected since they showed higher Slug expression as compared to those in Ishikawa and Hecc251 cells after culturing in STK2 (Fig. 1b). Cultured Hec6 cells had a low cell proliferation rate, particularly in the exponential growth phase, which correlated with increased p21^waf1^ but not p27^Kip1^ expression, and a decreased S-fraction during cell cycle progression (Fig. 2a). The inhibitory effects were also observed in Ishikawa and Hec251 cells (data not shown). Aldefluor assay revealed an increase in the ALDH1^high^ activity population (Fig. 2b), in line with the significantly increased number of well-defined, round spheroids that were over 50 um in size (Fig. 2c). In addition, expression of other CSC markers, including CD44s and Sox2, but not CD133, was also increased in Hec6 cells as well as Ishikawa and Hec251 cells cultured in STK2 (Additional file 1: Figure S1B). These findings indicated that the Em Ca cells cultured in STK2 exhibited EMT/CSC properties.

### Slug is associated with EMT/CSC properties in Em Ca cells

To examine whether Slug is directly linked to induction of EMT/CSC properties, two independent Hec6 cell lines stably overexpressing Slug (H6SL#8 and #21) were established. The stable cells underwent a dramatic change in morphology to fibroblast-like mesenchymal features with decreased E-cadherin expression, independent of Snail status (Fig. 2d). In H6SL#8 cells, the proliferative activity was extremely low, along with an inhibition of the S- to G2/M-phase during cell cycle progression (Fig. 2e). In addition, the ALDH1^high^ activity population was increased in the stable cells (Fig. 2f). However, changes in expression of Sox4, Sox7, and Sox9 were relatively minor in H6SL#8 cells as compared to the mock (Additional file 1: Figure S1C). These findings suggested that exogenous overexpression of Slug is sufficient in induction of EMT/CSC properties in Em Ca cells, independent of Sox factors.

### Transcriptional regulation of the Slug gene by Sox factors in Em Ca cells

**Fig. 3:**
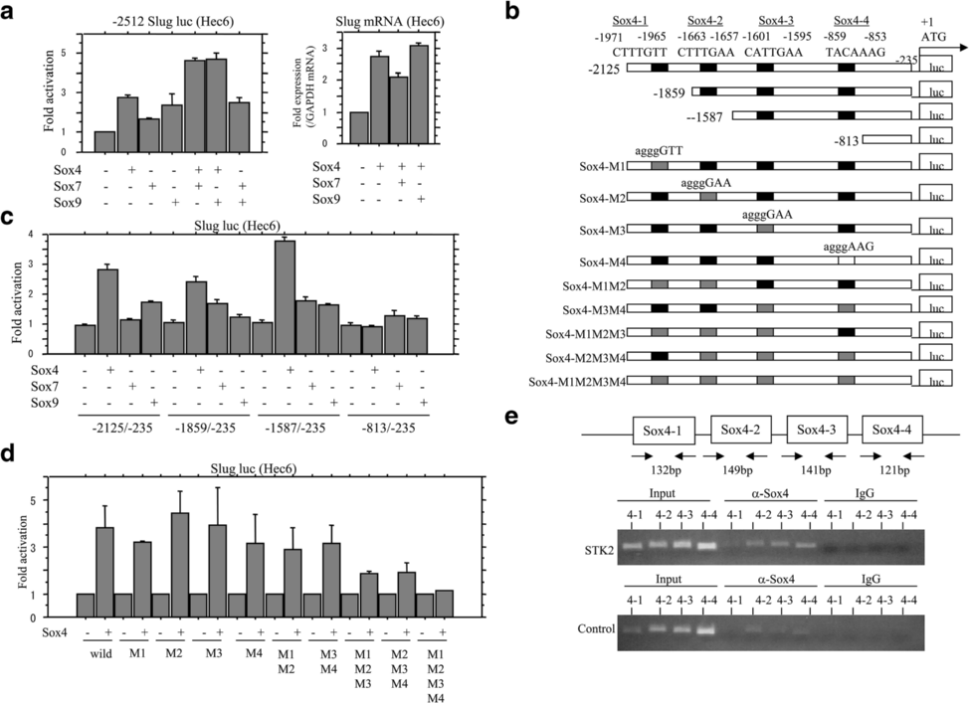
Transcriptional up-regulation of the *Slug* gene by Sox4. **a** *Left*: Hec6 cells were transfected with Slug reporter constructs, together with Sox4, Sox7, and Sox9, respectively. Relative activity was determined based on arbitrary light units of luciferase activity normalized to pRL-TK activity. The activities of the reporter plus the effector relative to that of the reporter plus empty vector are shown as means ± SDs. *Right*: analysis of mRNA levels for the *Slug* gene with total RNA extracted from Hec6 cells after transfection of Sox4, Sox7, and Sox9 using real time RT-PCR assays. The experiment was performed in triplicate. **b** The *Slug* promoter sequence containing four putative Sox4-binding sites including Sox4-1, Sox4-2, Sox4-3, and Sox4-4 sites. **c**, **d** Various promoter constructs were used for evaluating transcriptional regulation of the *Slug* promoter by Sox4, Sox7, and Sox9. The experiment was performed in triplicate. **e** ChIP assay data showing that Sox4 is bound to *Slug* promoter regions, in particular after culturing in STK2 for 3 days

To examine whether Sox factors are directly involved in transcription of the *Slug* gene, the three Sox factors, whose expression was differentially regulated by culturing in STK2, were transfected into three Em Ca cell lines. Transient transfection of Sox4 resulted in increased activity of the *Slug* promoter, along with increased mRNA levels, which were further enhanced by cotransfection of Sox7 or Sox9. In contrast, such effects were relatively minor when only Sox7 and/or Sox9 were transfected (Fig. 3a and Additional file 2: Figure S2A and B).

Analysis of an approximately 2000 bp fragment upstream of the translation start site in the *Slug* gene (AF084243) revealed four potential Sox4-binding elements (BE) (A/T A/T CAA A/T G) (Fig. 3b). Using a series of 5′-truncated promoter constructs, we found that deletion from −2125 to −1587 bp had little effect on induction of the promoter activity by Sox4, as well as Sox7 and Sox9, whereas the -813/-235 bp deletion appeared to have prevented binding of the Sox factors and reduced the promoter activity to a very low level (Fig. 3c and Additional file 2: Figure S2C). This indicated that the region involved in the response to Sox4 is present between −2125 to −813 bp. Additional promoter constructs carrying four nucleotide alterations in all of the putative Sox4-binding sites, including Sox4-1, Sox4-2, Sox4-3, and Sox4-4 (Fig. 3b), resulted in considerable reduction of response to Sox4 (Fig. 3d). ChIP assays revealed that increased amount of Sox4 by STK2 culture caused its recruitment to these four Sox4-BEs within the promoter (Fig. 3e). These findings suggest that *Slug* is a target gene of Sox4.

### Association between β-catenin and Sox4 on transcriptional regulation of the Slug gene in Em Ca cells

**Fig. 4:**
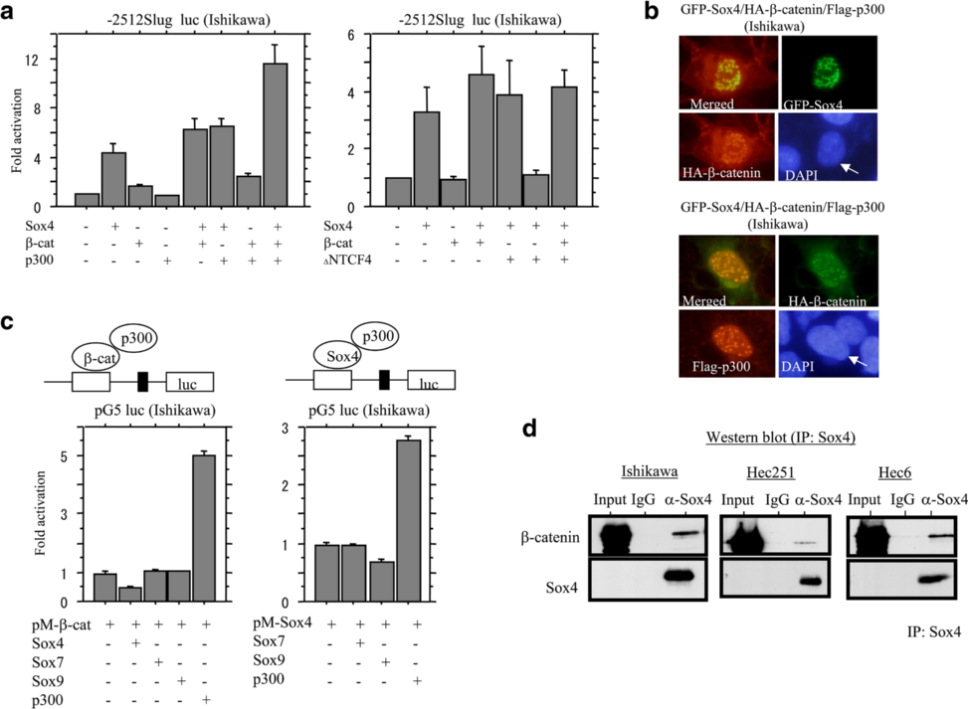
Interaction among β-catenin, Sox4, and p300 in Em Ca cells. **a** Ishikawa cells were transfected with Slug reporter constructs, together with β-cateninΔS45 (β-cat), Sox4, p300, and dominant-negative TCF4 (ΔNTCF4). Relative activity was determined based on arbitrary light units of luciferase activity normalized to pRL-TK activity. The activities of the reporter plus the effector relative to that of the reporter plus empty vector are shown as means ± SDs. The experiment was performed in duplicate. **b** After transfection of HA-β-cateninΔS45, GFP-Sox4, and Flag-p300, Ishikawa cell were stained with anti-HA antibody (*upper*) or a combination of anti-HA and anti-Flag antibodies (*lower*). Immunopositive cells are indicated by arrows. Nuclei were stained with DAPI. **c** Ishikawa cells were transfected with pM-β-catenin (*left*) or pM-Sox4 (*right*), along with pGL5 luc, Sox4, Sox7, Sox9, and p300. The experiment was performed in duplicate. **d** Coimmunoprecipitation of β-catenin and Sox4 in Ishikawa, Hec251, and Hec6 cells cultured in STK2 for 5 days. IP, immunoprecipitation

Since it has been reported that β-catenin is a positive regulator of Slug expression, [18] we examined for an association between Sox4 and β-catenin in regulation of the *Slug* promoter. The promoter activity was increased by a combination of β-catenin and Sox4, and the effect was further enhanced by cotransfection of the multifunctional coactivator p300, but not by dominant-negative TCF4 (Fig. 4a and Additional file 3: Figure S3A). Cotransfection of GFP-Sox4, HA-β-cateninΔS45, and Flag-p300 resulted in formation of several enlarged dots in the nuclei (Fig. 4b), whereas such nuclear aggregates were not observed after cotransfection of GFP-Sox4 and HA-β-cateninΔS45 alone (Additional file 3: Figure S3B). One-hybrid assays revealed that the pG5 luc reporter activity was raised by 3–12 folds following cotransfection of either DNA-BD-fused full-length β-catenin or Sox4 fragment (pM-β-cat or pM-Sox4) and p300, but such effects were relatively minor with cotransfection of only pM-β-catenin and Sox4, Sox7 or Sox9 (Fig. 4c and Additional file 3: Figure S3C and D). Finally, coimmunoprecipitation using lysates of cells cultured in STK2 revealed a weak interaction between β-catenin and Sox4 (Fig. 4d). These findings suggested that Sox4 cooperates with β-catenin/p300 complexes in the transcriptional regulation of the *Slug* gene.

### Immunohistochemical (IHC) findings in UCSs

**Fig. 5:**
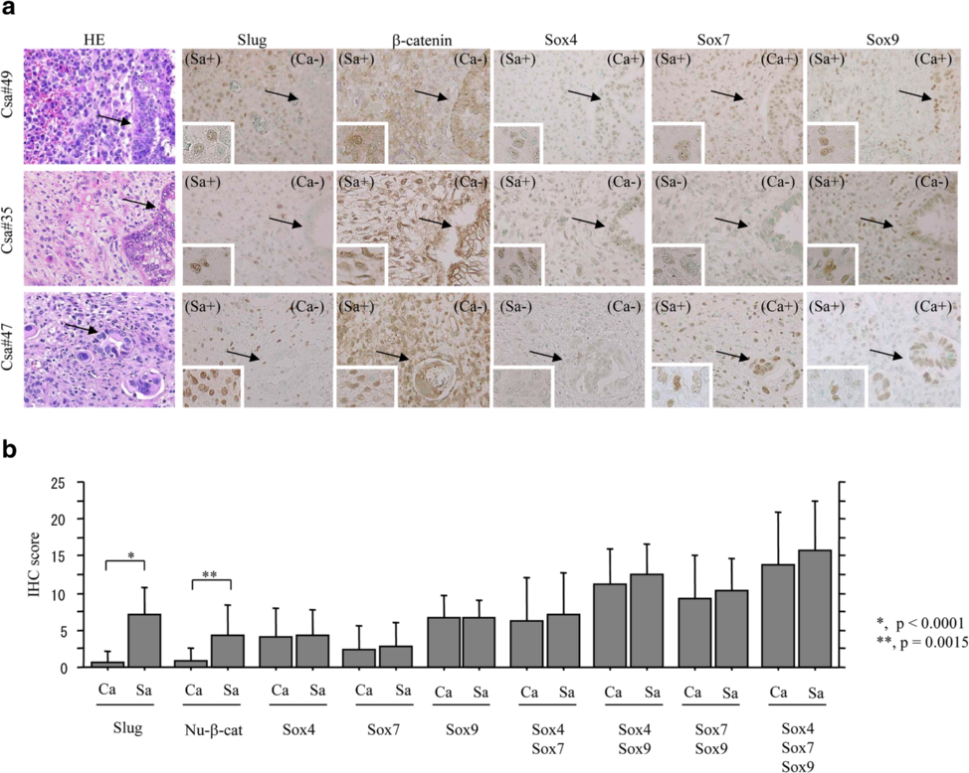
IHC findings in serial sections of UCS. **a** Staining by hematoxylin and eosin (HE) and by IHC for the indicated molecules. Note the predominant nuclear immunopositivity for Slug and β-catenin in sarcomatous components, but not carcinomatous lesions. Various combinations of nuclear immunopositivity for Sox4, Sox7, and Sox9 are also observed in both sarcomatous and carcinomatous components. Carcinomatous components are indicated by arrows. Sarcomatous components are magnified in the insets. Original magnification, x200 and x400 (insets). (Sa +/-), immunopositive or immunonegative in sarcomatous components; (Ca +/-), immunopositive or immunonegative in carcinomatous components. **b** IHC scores for the indicated molecules in carcinomatous (Ca) and sarcomatous (Sa) components of UCSs. The data shown are means ± SDs

Representative images of IHC findings for Slug, β-catenin, and Sox factors are illustrated in Fig. 5a. Distinct nuclear immunostaining for Slug, Sox4, Sox7, and Sox9, and nuclear and cytoplasmic/membranous immunoreaction for β-catenin were observed in both the carcinomatous and sarcomatous components of UCSs. Average nuclear β-catenin and Slug scores were significantly higher in the sarcomatous elements than those in the carcinomatous components, in contrast to no significant differences in the scores of Sox4, Sox7, and Sox9 (Fig. 5b). The average Slug score was positively correlated with nuclear β-catenin score and combinations of the three Sox factors, including Sox4/Sox9, Sox7/Sox9, and Sox4/Sox7/Sox9. The nuclear β-catenin score also showed a positive correlation with combinations of the three Sox factors (Table 2).

**Table 2:** Correlations among IHC markers investigated in uterine carcinosarcomas

	Slug	N-β-catenin	Sox4	Sox7	Sox9	Sox4 + Sox7	Sox4 + Sox9	Sox7 + Sox9
	*ρ* (p)	*ρ* (p)	*ρ* (p)	*ρ* (p)	*ρ* (p)	*ρ* (p)	*ρ* (p)	*ρ* (p)
N-β-catenin	0.59 (<0.0001)	*	*	*	*	*	*	*
Sox4	0.27 (0.08)	0.25 (0.1)	*	*	*	*	*	*
Sox7	0.3 (0.06)	0.46 (0.004)	0.42 (0.006)	*	*	*	*	*
Sox9	0.26 (0.22)	0.21 (0.07)	0.15 (0.48)	0.32 (0.14)	*	*	*	*
Sox4 + Sox7	0.3 (0.05)	0.32 (0.04)	0.87 (<0.0001)	0.78 (<0.0001)	0.3 (0.18)	*	*	*
Sox4 + Sox9	0.49 (0.03)	0.57 (0.009)	0.84 (<0.0001)	0.43 (0.04)	0.59 (0.004)	0.79 (0.0003)	*	*
Sox7 + Sox9	0.47 (0.05)	0.65 (0.005)	0.44 (0.04)	0.9 (<0.0001)	0.64 (0.003)	0.76 (0.0005)	0.58 (0.008)	*
Sox4 + Sox7 + Sox9	0.59 (0.01)	0.71 (0.001)	0.8 (0.0002)	0.82 (0.0002)	0.5 (0.02)	0.95 (<0.0001)	0.85 (0.0001)	0.87 (<0.0001)

*ρ* Spearman’s correlation coeffcient, *N* nuclear, *, not examined

## Discussion

The present study clearly provided evidence for a close link between EMT and CSC properties in Em Ca cells. We found that culturing Em Ca cells in STK2 was sufficient to induce EMT, as demonstrated by the acquirement of a spindle-like morphology as well as decreased E-cadherin and increased Slug expression. The IHC data also demonstrated a significantly higher Slug score in sarcomatous elements relative to carcinomatous components of UCSs. Further, the cell proliferation rates were significantly decreased during the process, in line with the report showing that Snail-expressing epithelial cells undergoing EMT have a low proliferation potential [24]. The cells cultured in STK2 also showed stem cell properties as evidenced by an increase in the ALDH1^high^ cell population and the number of spheroid formation. Given that EMT leads to a greater number of self-renewing cells that can initiate the seeding of spheroids with enriched stem cells, [14] it is likely that mesenchymal stem-like cells derived from carcinoma cells may be necessary for establishment of the sarcomatous components in UCSs.

In addition to EMT/CSC properties, the Em Ca cells cultured in STK2 also exhibited simultaneous upregulation of Sox4, Sox7, and Sox9. Interestingly, the *Sox4* promoter activity was drastically increased by transfection of Sox7, in line with the IHC data showing significant positive correlation between the two in UCS tissues. Although both *Sox7* and *Sox9* promoters were also weakly activated by either Sox4, Sox7, or Sox9 in Hec6 cells, such associations were absent in UCS tissues. Given the evidence that expression of *Sox* genes themselves is frequently subjected to auto-regulation or control by other Sox proteins, [25] it appeared that complex regulatory loops among these Sox factors including the Sox7/Sox4 axis may be activated during the EMT/CSC process.

Unexpectedly, we found that Hec6 cells stably overexpressing Slug did not show any changes in expression of Sox4, Sox7, and Sox9, although the cells displayed EMT/CSC properties. This may be because Sox factors are upstream of Slug and thus are no longer required for the process in cells exogenously overexpressing Slug. In addition, it is also unexpected that there were no significant differences in the Sox factor scores between sarcomatous and carcinomatous components in UCSs, although combinations of the Sox factors showed positive correlations with both Slug and nuclear β-catenin scores. At the present time, although we are unable to provide an appropriate explanation for the observation, one possible reason may be that various post-translational modifications modulate the activity, stability, and intracellular localization of some Sox proteins. For example, some Sox factors are subject to various covalent modifications such as phosphorylation, sumoylation, acetylation, methylation, and glycosylation [26–31]. Further studies to clarify these points are clearly warranted.

Several lines of evidence from the present study support the conclusion that Sox4 contributes to transcriptional control of the *Slug* gene. First, overexpression of both Slug and Sox4 occurred in Em Ca cells cultured in STK2. Second, transient transfection of Sox4 caused an increase in Slug mRNA expression, in line with activation of its promoter. Third, Sox4 could bind to the promoter region of the *Slug* gene from −2125 to −813 bp, probably through its interaction with four putative Sox4-BEs. The effects were further enhanced by cotransfection of Sox9 and/or Sox7. Interestingly, it has been recently reported that ectopic Sox4 expression in human mammary epithelial cells could induce a mesenchymal phenotype, which was associated with increased stem cell properties, cellular migration, and invasion in vitro [32].

Sox proteins generally exhibit gene regulatory functions only by forming complexes with partner transcription factors [25]. Transcriptional activity and target gene specificity of Sox4 are also considered to be controlled through cooperative interactions with distinct transcription factors and cofactors [33]. In this study, Sox4 was able to enhance β-catenin-mediated transcription of the *Slug* gene through formation of transcriptional complexes including β-catenin, Sox4, and p300. Although Sox4 has been demonstrated to directly interact with TCF4 via their respective HMG domain by *in vitro* protein-binding assay, [34] our present data revealed that activation of the *Slug* promoter by Sox4, as well as β-catenin, was not abrogated by dominant-negative TCF4, indicating that the observed activation did not require TCF4-binding sites in the promoter.

## Conclusions

**Fig. 6:**
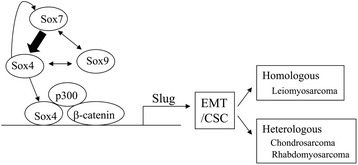
Schematic representation of the association between Sox factors and β-catenin/p300 signal networks in the establishment of EMT/CSC properties as progenitor for divergent sarcomatous differentiation in UCS

Our observations suggest a mode of molecular mechanism for establishment of sarcomatous components with EMT/CSC properties in UCSs (Fig. 6). Upregulation of Slug by Sox4, as well as β-catenin and p300 complexes, induces EMT and associated CSC properties, which in turn results in the promotion of homologus and heterologous sarcomatous components in UCSs. Increased expression of Sox7 and Sox9, as well as cooperation between Sox7 and Sox4, also participate in the process. Thus, the present study clearly provided evidence of a functional role for Sox4 through its association with β-catenin and p300 in regulation of the *Slug* gene during development of the sarcomatous component in UCSs.

## Additional files

Additional file 1: Figure S1. (A) Ishikawa (Ish) cells were transfected with Sox4, Sox7, and Sox9 reporter constructs, together with either Sox4, Sox7, or Sox9. Relative activity was determined based on arbitrary light units of luciferase activity normalized to pRL-TK activity. The activities of the reporter plus the effector relative to that of the reporter plus empty vector are shown as means ± SDs. The experiment was performed in duplicate. (B) Western blot analysis for the indicated proteins from Ishikawa, Hec251, and Hec6 cells cultured in STK2. (C) Western blot analysis for the indicated proteins from H6SL#8 and mock cells. (TIF 2816 kb) Additional file 2: Figure S2. (A) Ishikawa (left) and Hec251 cells (right) were transfected with Slug reporter constructs, together with Sox4, Sox7, and Sox9. Relative activity was determined based on arbitrary light units of luciferase activity normalized to pRL-TK activity. The activities of the reporter plus the effector relative to that of the reporter plus empty vector are shown as means ± SDs. (B) Analysis of mRNA levels for the *Slug* gene with total RNA extracted from Hec251 cells after transfection of Sox4, Sox7, and Sox9 using real time RT-PCR assays. The experiment was performed in triplicate. (C) Various promoter constructs were used for evaluating transcriptional regulation of the *Slug* promoter by Sox4, Sox7, and Sox9 in Ishikawa (left) and Hec251 cells (right). The experiment was performed in triplicate. (TIF 1198 kb) Additional file 3: Figure S3. (A) Hec251 (left) and Hec6 cells (right) were transfected with Slug reporter constructs, together with β-cateninΔS45 (β-cat), Sox4, and p300. Relative activity was determined based on arbitrary light units of luciferase activity normalized to pRL-TK activity. The activities of the reporter plus the effector relative to that of the reporter plus empty vector are shown as means ± SDs. The experiment was performed in duplicate. (B) After transfection of HA-β-cateninΔS45 and GFP-Sox4, cells were stained with anti-HA antibody. Immunopositive cell is indicated by arrow. Nuclei were stained by DAPI. (C,D) Hec251 (C) and Hec6 (D) cells were transfected with pM-β-catenin (left) or pM-Sox4 (right), along with pGL5 luc, Sox4, Sox7, Sox9, and p300. The experiment was performed in duplicate. (TIF 2017 kb)

## Abbreviations

ALDH1: aldehyde dehydrogenase 1
CSC: cancer stem cell
EMT: epithelial-mesenchymal transition
UCS: uterine carcinosarcoma
