# Supplementary figures and images for: Cooperation of Sox4 with β-catenin/p300 complex in transcriptional regulation of the Slug gene during divergent sarcomatous differentiation in uterine carcinosarcoma
Source: BMC Cancer. 2016 Feb 3;16:53. doi: 10.1186/s12885-016-2090-y (PMC4739330; doi:10.1186/s12885-016-2090-y)

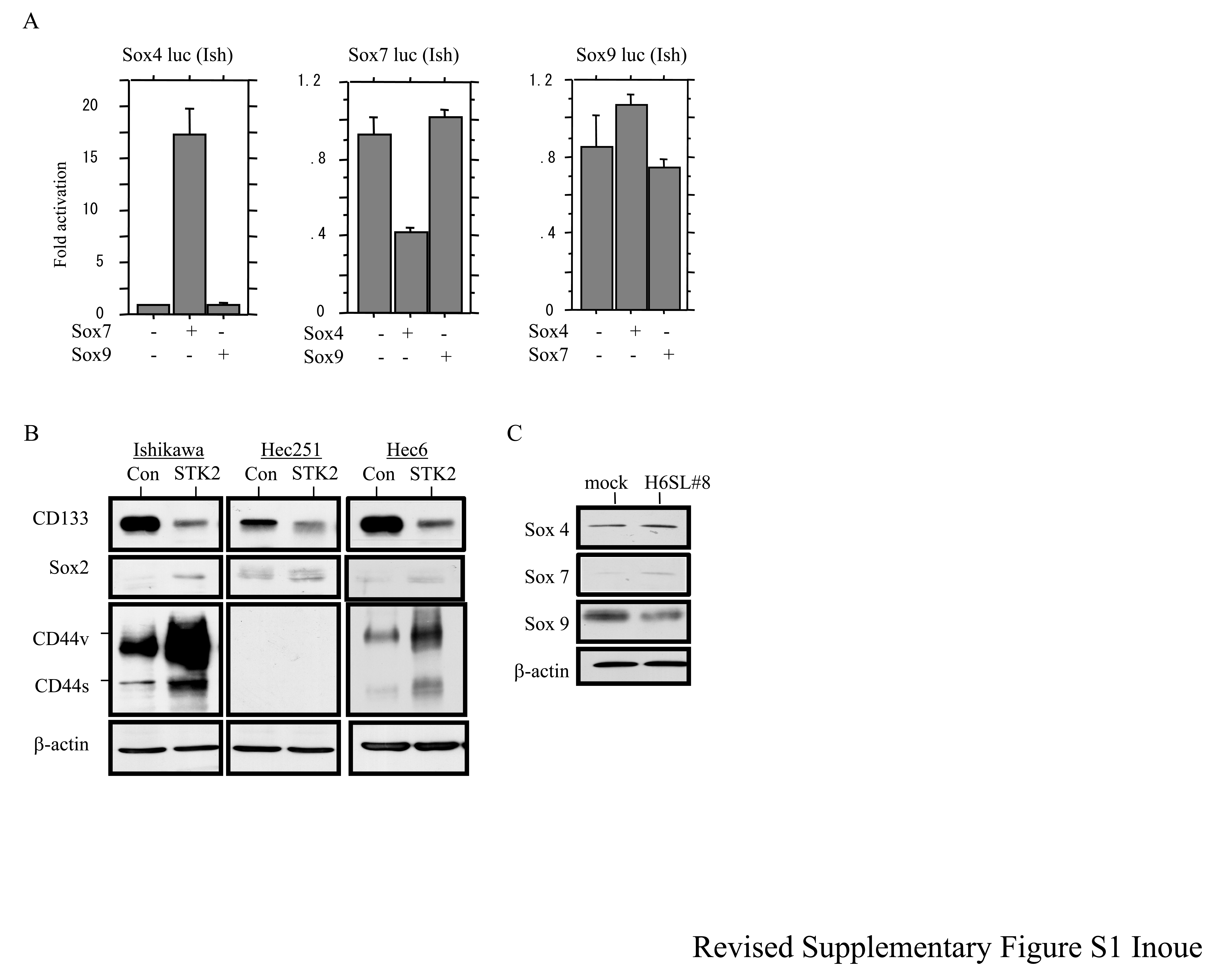

Supplement: Additional file 1: Figure S1. — (A) Ishikawa (Ish) cells were transfected with Sox4, Sox7, and Sox9 reporter constructs, together with either Sox4, Sox7, or Sox9. Relative activity was determined based on arbitrary light units of luciferase activity normalized to pRL-TK activity. The activities of the reporter plus the effector relative to that of the reporter plus empty vector are shown as means ± SDs. The experiment was performed in duplicate. (B) Western blot analysis for the indicated proteins from Ishikawa, Hec251, and Hec6 cells cultured in STK2. (C) Western blot analysis for the indicated proteins from H6SL#8 and mock cells. (TIF 2816 kb) [file 12885_2016_2090_MOESM1_ESM.tif]

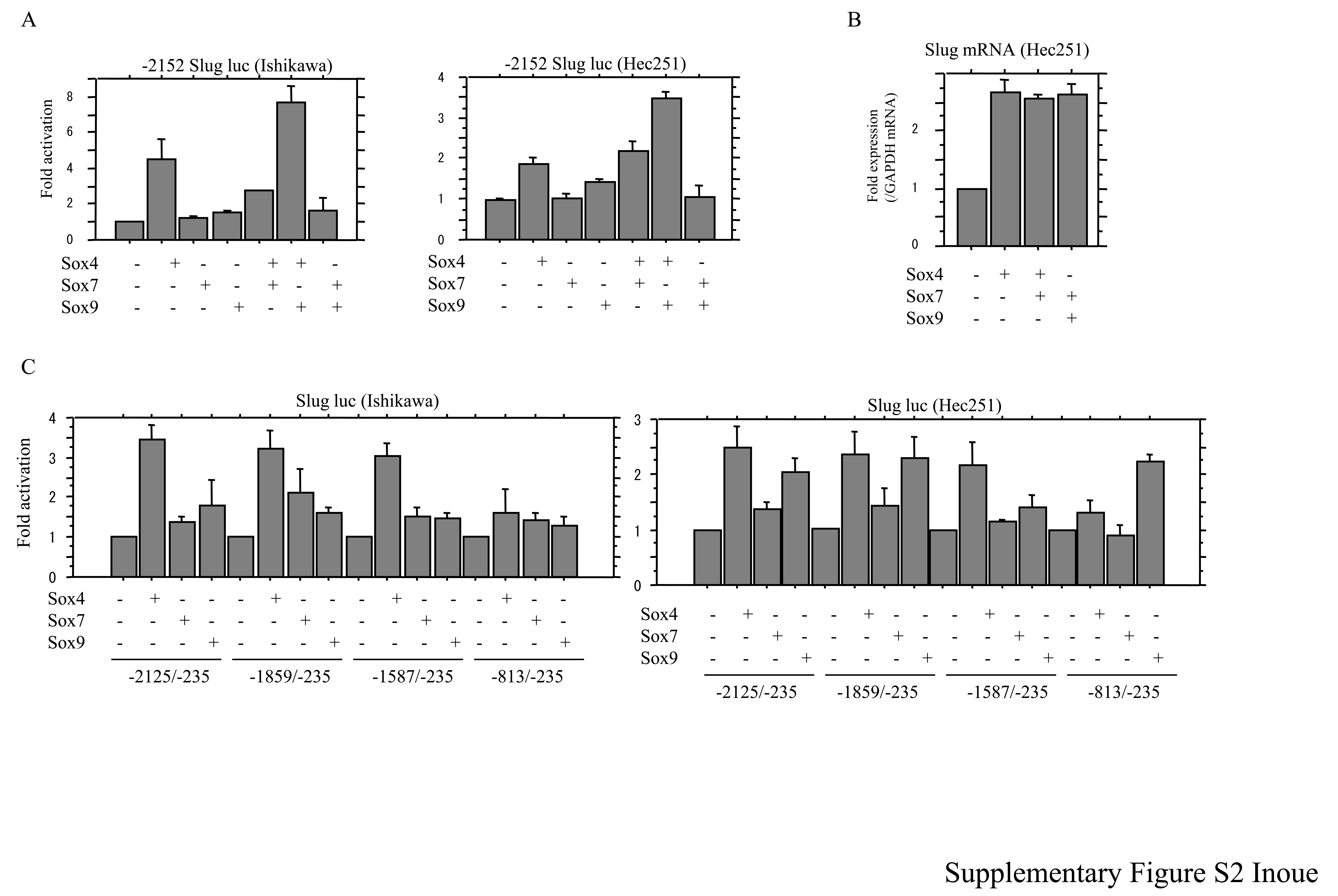

Supplement: Additional file 2: Figure S2. — (A) Ishikawa (left) and Hec251 cells (right) were transfected with Slug reporter constructs, together with Sox4, Sox7, and Sox9. Relative activity was determined based on arbitrary light units of luciferase activity normalized to pRL-TK activity. The activities of the reporter plus the effector relative to that of the reporter plus empty vector are shown as means ± SDs. (B) Analysis of mRNA levels for the Slug gene with total RNA extracted from Hec251 cells after transfection of Sox4, Sox7, and Sox9 using real time RT-PCR assays. The experiment was performed in triplicate. (C) Various promoter constructs were used for evaluating transcriptional regulation of the Slug promoter by Sox4, Sox7, and Sox9 in Ishikawa (left) and Hec251 cells (right). The experiment was performed in triplicate. (TIF 1198 kb) [file 12885_2016_2090_MOESM2_ESM.tif]

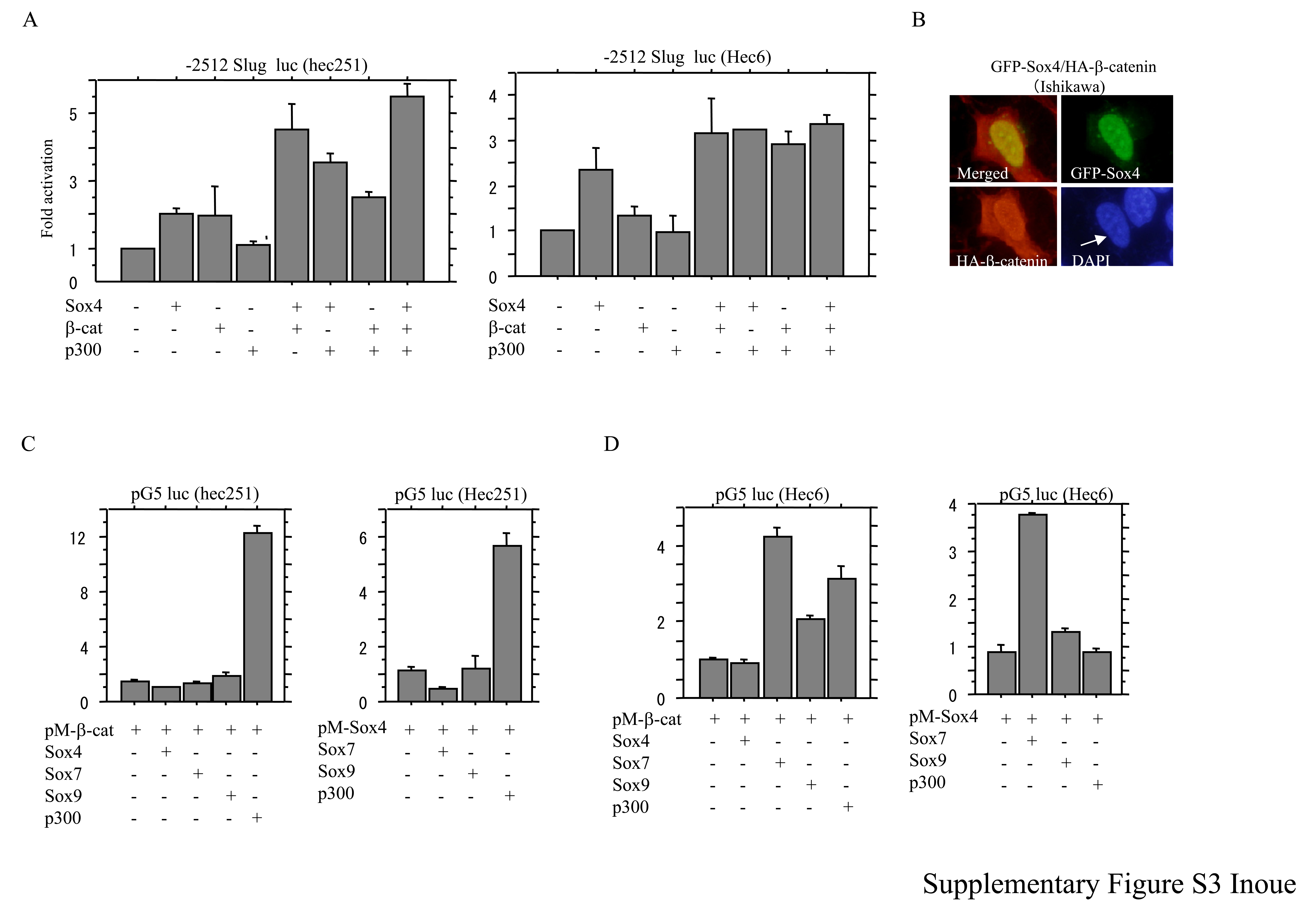

Supplement: Additional file 3: Figure S3. — (A) Hec251 (left) and Hec6 cells (right) were transfected with Slug reporter constructs, together with β-cateninΔS45 (β-cat), Sox4, and p300. Relative activity was determined based on arbitrary light units of luciferase activity normalized to pRL-TK activity. The activities of the reporter plus the effector relative to that of the reporter plus empty vector are shown as means ± SDs. The experiment was performed in duplicate. (B) After transfection of HA-β-cateninΔS45 and GFP-Sox4, cells were stained with anti-HA antibody. Immunopositive cell is indicated by arrow. Nuclei were stained by DAPI. (C,D) Hec251 (C) and Hec6 (D) cells were transfected with pM-β-catenin (left) or pM-Sox4 (right), along with pGL5 luc, Sox4, Sox7, Sox9, and p300. The experiment was performed in duplicate. (TIF 2017 kb) [file 12885_2016_2090_MOESM3_ESM.tif]
